# Gibson assembly: an easy way to clone potyviral full-length infectious cDNA clones expressing an ectopic VPg

**DOI:** 10.1186/s12985-015-0315-3

**Published:** 2015-06-14

**Authors:** Amandine Bordat, Marie-Christine Houvenaghel, Sylvie German-Retana

**Affiliations:** INRA, UMR 1332 de Biologie du Fruit et Pathologie, CS 20032, 33882 Villenave d’Ornon, France; Université de Bordeaux, UMR 1332 de Biologie du Fruit et Pathologie, CS 20032, 33882 Villenave d’Ornon, France

**Keywords:** Full-length infectious clone, Gibson assembly, Potyvirus, VPg, Improved cloning

## Abstract

**Background:**

Approaches to simplify and accelerate the construction of full-length infectious cDNA clones for plant potyviruses have been described, based on cloning strategies involving *in vitro* ligation or homologous recombination in yeast. In the present study, we developed a faster and more efficient *in vitro* recombination system using Gibson assembly (GA), to engineer a *Lettuce mosaic virus* (LMV) infectious clone expressing an ectopic mcherry-tagged VPg (Viral protein genome-linked) for *in planta* subcellular localization of the viral protein in an infection context.

**Methods:**

Three overlapping long distance PCR fragments were amplified and assembled in a single-step process based on *in vitro* recombination (Gibson assembly). The resulting 17.5 kbp recombinant plasmids (LMVmchVPg_Ec) were inoculated by biolistic on lettuce plants and then propagated mechanically on *Nicotiana benthamiana*. Confocal microscopy was used to analyze the subcellular localization of the ectopically expressed mcherry-VPg fusion protein.

**Results:**

The Gibson assembly allowed the cloning of the expected plasmids without any deletion. All the inoculated plants displayed symptoms characteristic of LMV infection. The majority of the mcherry fluorescent signal observed using confocal microscopy was located in the nucleus and nucleolus as expected for a potyviral VPg.

**Conclusions:**

This is the first report of the use of the Gibson assembly method to construct full-length infectious cDNA clones of a potyvirus genome. This is also the first description of the ectopic expression of a tagged version of a potyviral VPg without affecting the viability of the recombinant potyvirus.

**Electronic supplementary material:**

The online version of this article (doi:10.1186/s12985-015-0315-3) contains supplementary material, which is available to authorized users.

## Background

The availability of full-length infectious cDNA clones (FL-cDNAs) is crucial for reverse genetics studies on plant viruses. Since the first description of the cloning of an infectious clone of *Brome mosaic virus* [1], many other infectious clones of plant RNA viruses were obtained by cloning the full-length genomic cDNA under T7 RNA polymerase or 35S promoter sequences [2–4]. Approaches to simplify and accelerate the construction of FL-cDNAs for plant viruses were recently described, based on cloning strategies involving homologous recombination in yeast [3, 5] rather than more classical cloning approaches such as *in vitro* ligation. Furthermore, in the last few years, various methods for assembling large DNA molecules have been developed [6]. *In vitro* assembly and yeast-based *in vivo* method were used in tandem to assemble the whole bacterial genome of *Mycoplasma genitalium* [7]. The development of *in vitro* methods allowed the synthesis of the first chemical mouse mitochondrial genome [8] using the isothermal assembly method called Gibson assembly [9]. This technique was further applied to construct animal viral genomes such as *Dengue virus* (DENV) [10] *West nile virus* (WNV) [11] and *Porcine reproductive and respiratory syndrome virus* (PRRSV) [12]. Animal viral genomes were even assembled *de novo* following chemical synthesis in the absence of natural template [13]. In the case of plant viruses, the development of synthetic cDNA clones was recently described for two members of the plant virus genera *Tobamovirus* and *Tombusvirus* [14, 15].

In this paper, we report the development of a rapid and efficient *in vitro* recombination system based on assembling overlapping PCR-amplified DNA molecules in a single isothermal step, derived from the strategy based on *in vitro* Gibson assembly (GA) [9]. This strategy was used to obtain modified infectious cDNA clones of a potyvirus, *Lettuce mosaic virus* (LMV). The genus *Potyvirus* is the largest genus of plant viruses, causing considerable economic losses in various crops [16, 17]. Potyviruses have flexuous filamentous particles containing a positive single-stranded RNA, covalently linked to a viral protein genome-linked (VPg) at its 5′ end. The genome encodes a large polyprotein processed by three self-encoded proteinases to yield the mature viral proteins [17]. In recent years, components of the eukaryotic translation initiation complex were identified as essential determinants in the outcome of potyviral infection [18]. In particular, we have shown that the recessive resistance to LMV is conferred by the lettuce *mo1* gene, encoding the eukaryotic translation initiation factor 4E (eIF4E) [19–21]. We demonstrated that the VPg together with the Cylindrical inclusion helicase (CI) are directly involved in overcoming *mo1* resistance [22, 23] and in binding to lettuce eIF4E [24]. Such interactions should play crucial roles in many processes during the virus infection cycle, and probably during the eIF4E recruitment process.

In the context of deciphering the contribution of the VPg protein to lettuce-LMV interactions we aimed at engineering LMV FL-cDNA clones expressing a mcherry-tagged VPg (LMVmchVPg), in order to perform *in planta* subcellular localization of this viral protein in infected cells. For that purpose, we initially engineered a recombinant LMV expressing the mcherry protein fused to either the N-terminal or the C-terminal ends of the VPg, using homologous recombination in yeast to assemble overlapping DNA fragments. Unfortunately, LMV variants with deletions of the inserted mcherry gene were rapidly selected *in planta*, showing that the mcherry-tagging of the VPg significantly affected the fitness of LMV (*data not shown*). To solve this problem, we engineered LMV recombinants expressing an ectopic form of mcherry-tagged VPg in addition of the native VPg (LMVmchVPg_Ec), using the Gibson assembly method [9]. This is the first report of a recombinant potyvirus infectious cDNA clone obtained using Gibson assembly, as well as the first description of the ectopic expression of a potyviral VPg without affecting the viability of the recombinant potyvirus vector.

## Results and discussion

### Construction of an LMV FL-cDNA clone expressing a mcherry-tagged VPg using Gibson assembly method

**Fig. 1:**
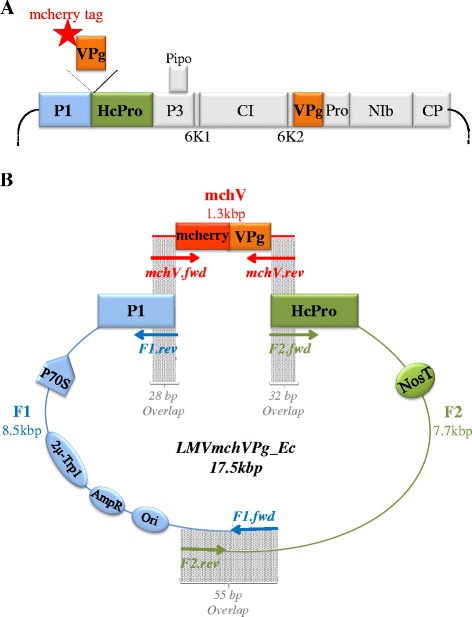
Cloning of LMVmchVPg_Ec full-length cDNA by *in vitro* recombination Gibson assembly strategy. **a** Schematic representation of the infectious clone LMVmchVPg_Ec genome. The ectopically expressed mcherry-VPg fusion protein gene is inserted between the P1 and HcPro sequences of the *Lettuce mosaic virus* (LMV) genome (red star: mcherry fluorescence tag fused at the N-terminus of the VPg). **b** Schematic representation of the cloning by *in vitro* recombination strategy. The size of the overlapping regions between the PCR fragments where recombination takes place is indicated. The three overlapping fragments named mchV, F1 and F2 (highlighted in red, blue and green respectively) were amplified by long distance PCR using the primers pairs (arrows) positioned along the cloning vector or the viral LMV template. The full-length cDNA of LMV is derived from the LMV-0 cDNA clone previously reported by Redondo *et al*. [30] and further modified by Sorel *et al.* [23]. The LMV FL- cDNA was assembled into a pBluescribe-derived vector pBS70T containing an enhanced 35S promoter (P70S), a NOS terminator (NosT), an *E.coli* replication origin (Ori), an ampicillin resistance gene (AmpR), a cassette containing the 2 μ yeast replication origin and a yeast selectable marker (Trp-1promoter and gene, 2 μ-Trp1).The three PCR products were assembled *in vitro* by Gibson assembly. bp: base pair; kbp: kilobase pair

**Fig. 2:**
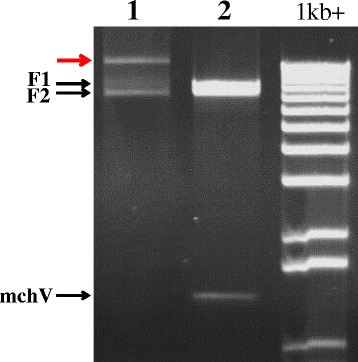
Agarose gel electrophoresis analysis of the overlapping fragments and the resulting *in vitro* recombination product. Analysis on a 0.8 % non-denaturing agarose gel of the three PCR amplification products in the absence of Gibson assembly mix (**lane 2**, negative control, incubation in water 1 h at 50 °C) and after recombination by Gibson assembly (**lane 1,** incubation in Gibson Assembly Master Mix 1 h at 50 °C). The black arrows indicate the position of each PCR amplification product. The red arrow indicates the position of the recombination product obtained after Gibson assembly. 1 kb+: 1 Kb Plus DNA Ladder (Invitrogen)

The genome of the final expected construct, named LMVmchVPg_Ec, is represented on Fig. 1a. The mcherry sequence is fused at the N-terminus of the VPg and the mcherry-VPg fusion gene is cloned between the P1 and HC-Pro coding sequences, at the cloning site currently used for LMV engineering [25, 26]. Indeed, tag insertions between NIb and CP were tried for LMV but led to a decrease in LMV infection (unpublished results). In an effort to preserve the biological properties of the parental LMV isolate, an artificial cleavage site for the NIa viral proteinase (DEVYHQ/SG) was inserted at the end of the ectopic mcherry-VPg sequence, allowing the recovery of an unfused HC-Pro protein following proteolytic maturation of the polyprotein [26]. The cloning strategy used to assemble this construct is described in Fig. 1b. Two divergent overlapping PCR fragments (F1 and F2) of respectively 8.5 kbp and 7.7 kbp, corresponding to the LMV infectious cDNA and to the cloning vector , were amplified from a LMV cDNA clone described in a previous study [23] and derived from the LMV-0 isolate [GenBank: X9770]. The F1 and F2 fragments were respectively amplified with the primers pairs F1.fwd/F1.rev and F2.fwd/F2.rev (Table 1). The mcherry-VPg (mchV) sequence was amplified from LMVmchVPg template (Additional files 1 and 2) with the primers mchV.fwd and mchV.rev listed in Table 1. The three purified overlapping fragments (F1, F2 and mchV) were further assembled in one single-step reaction by GA. The efficiency of the GA *in vitro* recombination reaction was evaluated by agarose gel electrophoresis analysis (Fig. 2). The red arrow points out the high-molecular weight fragment corresponding to the recombination product obtained after the GA reaction (Fig. 2, lane 1). The majority of the PCR fragments disappeared after the GA reaction (compare Fig. 2 lane 1 and lane 2, negative control). In our experience, a key point for Gibson assembly strategy is to design the overlapping zone of the PCR amplified fragments. Indeed, during the GA reaction, the T5 exonuclease removes nucleotides from the 5′ end of each PCR fragment, releasing single stranded ends that can form secondary structure, such as hairpins. This may impair the annealing of the overlapping fragments and therefore reduce the efficiency of the *in vitro* recombination. Therefore, the position of the primers for long distance PCR amplification must be carefully chosen to avoid this situation.

**Table 1:** List of primers used for LMVmchVPg_Ec cloning and RT-PCR amplification

Primer name	Primer size (nt)	Amplified fragment	Sequence 5′-3′	Product size (kbp)
F1.fwd	30	F1	GCGGATCCAAAGGATAATATCATCACAGAG	8.5
F1.rev	28		**GTATTGAACCATACGGTGCGTGATGGAG**	
F2.fwd	49	F2	***GACGAAGTATACCACCAGTCCGGA*** **GACGTCGCA**CGAAACTTCTGGAACG	7.7
F2.rev	23		CTTCTTCATCTGCCCAGAACCAC	
mchV.fwd	58	mchV	**CTCCATCACGCACCGTATGGTTCAATAC**AGTGATGTAGTGAGCAAGGGCGAGGAGGAT	1.3
mchV.rev	52		**TGCGACGTCTCCGGACTGGTGGTATACTTCGT**CTTCGTGCTTTACAGGGATG	
P1Hc.fwd	20	3′P1-mchVPg-5′HcPro	GGCGACACAGTATATTCGTT	1.9
P1Hc.rev	21		TCACTAAAGTCATTCAGGAAC	

The regions of homology allowing *in vitro* recombination between fragments are indicated in bold. The artificial NIa cleavage site is indicated in italics. The primers P1Hc.fwd and P1Hc.rev were used to perform RT-PCR to confirm the presence of the mcherry-VPg gene expression in the viral progeny. The suffixes “fwd” indicate sense primers, while suffixes “rev” indicated antisense primers. *nt* nuclotides, *bp* base pair

After transformation of the recombination product in *E.coli* cells, 30 colonies were obtained. Plasmid was extracted from 24 colonies and restriction enzyme analysis showed that 12 colonies contained plasmids with the expected restriction profile. Two independent LMVmchVPg_Ec clones, LV14 and LV15, were chosen for further analysis.

### Infectivity of LMVmchVPg_Ec clones in lettuce plants

**Fig. 3:**
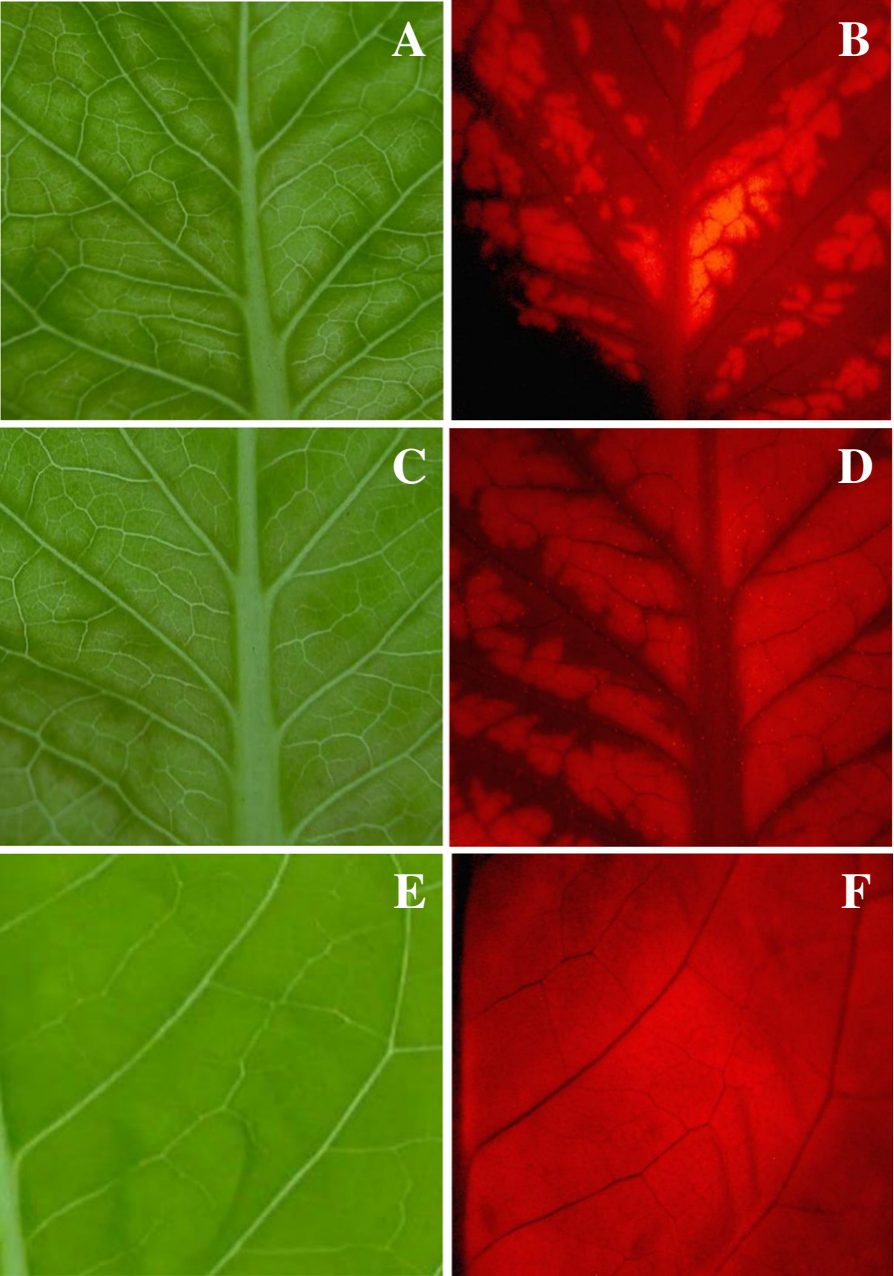
Symptoms induced by LMVmchVPg_Ec on the lettuce cultivar Trocadéro under white and UV light. The mcherry-VPg expression in systemically infected leaves, was examined by fluorescence microscopy with a 0.8× magnification, using a Leica MZFLIII Fluorescence Stereomicroscope. **a–d**: Plants infected with LMVmchVPg_Ec (clone LV15) at 15 days post inoculation (dpi) under white light (**a, c**) and UV light (**b, d**)**.** The fluorescence is superimposable to yellow-green areas of the mosaic symptoms pattern observed under white light. **e–f**: mock-inoculated Trocadéro plants under white light (**e**) and UV light (**f**)

Purified DNA of the LMVmchVPg_Ec clones LV14 and LV15 were coated on gold particles and inoculated by particle bombardment on six plants each of the susceptible lettuce cultivar Trocadéro. At 15 days after inoculation (dpi), all the inoculated plants displayed the characteristic vein clearing and mosaic symptoms of LMV infection on the systematically infected leaves (Additional file 3). The expression of the mcherry-VPg fusion was first examined in systemic leaves using a fluorescence stereomicroscope. Under UV light, the fluorescence signal (Fig. 3b, d) overlapped with the mosaic symptoms pattern observed under white light (Fig. 3a, c) confirming the replication and systemic propagation of both LMVmchVPg_Ec clones. The presence of the recombinant virus was confirmed by reverse transcription-PCR using LMV-specific primers P1Hc.fwd and P1Hc.rev (Table 1) targeting the insertion site of the ectopically mcherryVPg fusion (Additional file 4).

### Subcellular localization of mcherry-VPg fusion protein in infected leaves

**Fig. 4:**
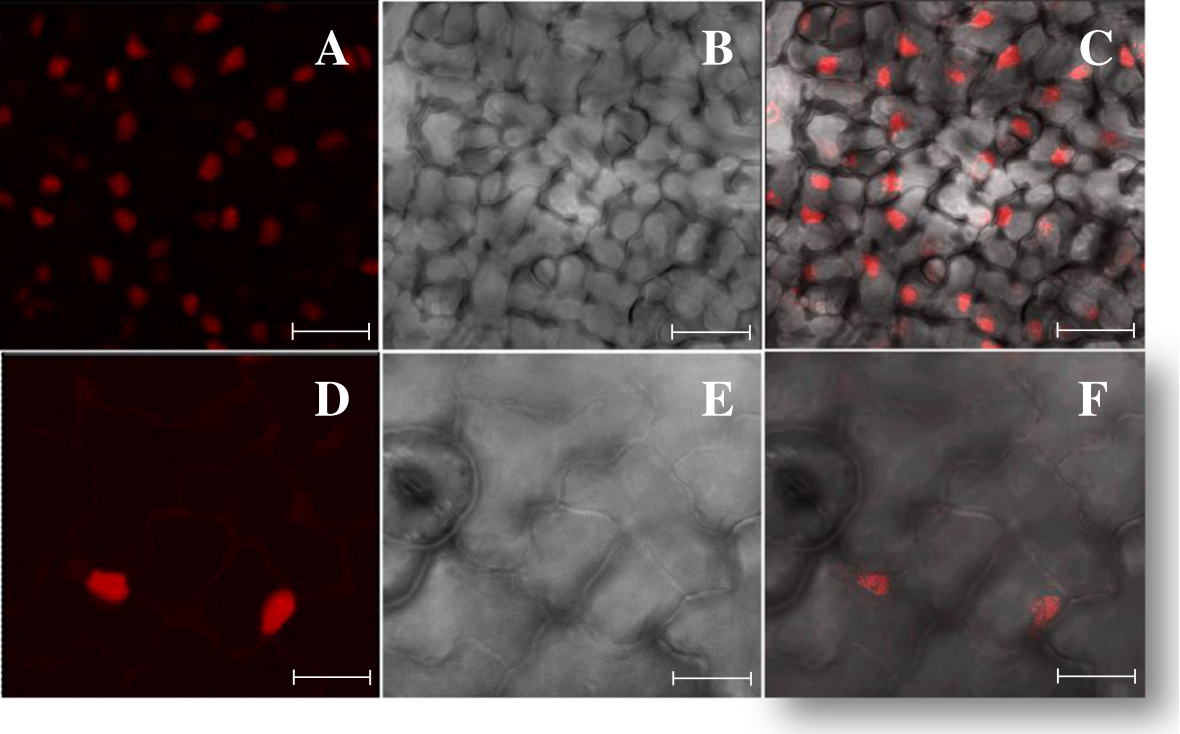
Detection of fluorescence emitted by the fusion protein mcherry-VPg in LMVmchVPg_Ec infected lettuce leaves. Fluorescence signals observed in the epidermal cells of the lettuce cultivar Trocadéro by confocal microscopy. Images for fluorescence emitted by mcherry-VPg at 20 dpi (**a, d**). Bright field images of the same cells (**b, e**) and corresponding overlay images (**c, f**). Scale bars: 27.39 μM (**a,b,c**), 15 μM (**d,e,f**)

**Fig. 5:**
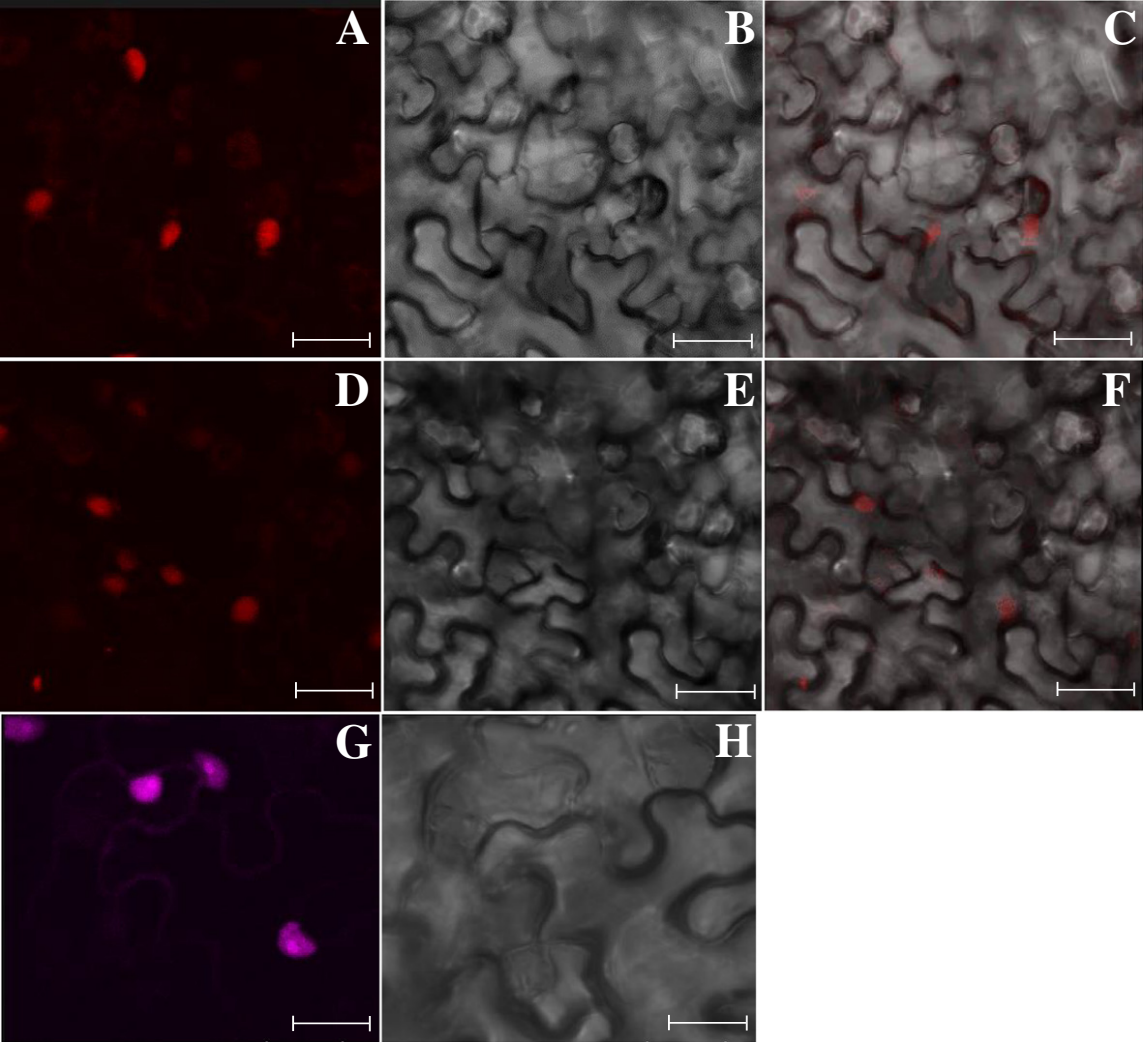
Detection of fluorescence emitted by the fusion protein mcherry-VPg in LMVmchVPg-Ec infected Nicotiana benthamiana leaves. Fluorescence signals observed in the epidermal cells of *Nicotiana benthamiana* by confocal microscopy. Images for fluorescence emitted by mcherry-VPg at 16 dpi (**a, d, g**). Bright field images of the same cells (**b, e, h**) and corresponding overlay images (**c, f**). The fluorescence signal in the nucleolus is particularly strong in the nuclei observed in **g**. Scale bars: 27.23 μM (**a,b,c**), 28 μM (**d,e,f**), 18.9 μM (**g,h**)

The subcellular localization of the mcherry-VPg fusion expressed in lettuce and in *N. benthamiana* (another LMV host currently used for transient expression of tagged protein by agroinfiltration), was further analyzed using confocal microscopy. At 20 dpi, the majority of the fluorescence signal was found in the nucleus and nucleolus of the infected lettuce cells (Fig. 4) or infected *N. benthamiana* cells (Fig. 5). The localization of the VPg to the nucleus was confirmed by DAPI staining of nuclear DNA (Additional file 5). The nuclear VPg localization was already observed for other potyviral VPg [27]. In particular, in 2009, Rajamaki and Valkonen showed that the VPg of *Potato virus A* accumulates in the nucleolus and interacts with fibrillarin [28]. They also showed that a bipartite nuclear localization signal (NLS) sequence mediates nuclear/nucleolar localization of the VPg which is crucial for viral replication. Concerning LMV, VPg residues two to eight (KGKRQRQ) are predicted to form a NLS in plant cells, according to functional prediction of the Eukaryotic Linear Motif (ELM) data base (http://elm.eu.org/) [29]. In this study, the subcellular localization of the mcherry-VPg ectopically expressed from LMV genome is therefore in good agreement with previous results and confirms that the mcherry-fusion does not alter the subcellular localization of the VPg. The recombinant LMVmchVPg_Ec clone will be useful to investigate the subcellular localization of the interactions between the VPg and its viral partners during LMV infection. In particular, the CI protein will be transiently expressed by agroinfiltration as a GFP-fusion protein in LMVmchVPg_Ec infected plants. The observation of the GFP and mcherry signals by confocal microscopy will allow us to precisely place where the VPg-CI interaction takes place.

## Conclusions

Full-length infectious cDNA clones are crucial for reverse genetics studies on plant RNA viruses. In this paper, we describe a significant methodological advance to simplify and accelerate the construction of infectious cDNA clones derived from the potyvirus *Lettuce mosaic* virus as a model example. This approach relies on an *in vitro* recombination system called Gibson assembly (GA), based on assembling and repairing overlapping PCR-amplified DNA molecules in a single isothermal step (Additional file 6). In our opinion, this constitutes proof of concept of the use of Gibson assembly to create a recombinant potyvirus genome. Using this GA-based strategy, we modified the LMV genome in order to ectopically express a mcherry-tagged VPg fusion protein during viral infection. We showed that this modification did not affect the viability of the recombinant potyvirus in contrast to direct tagging of the VPg. The mcherry-VPg fusion protein was observed in the nucleus and nucleolus of infected cells as expected, confirming that the fusion did not affect the subcellular localization of the VPg. The recombinant LMVmchVPg_Ec clone should prove a very interesting tool to further investigate the subcellular localization of the VPg and its viral and cellular partners during LMV infection.

## Methods

### Full-length cDNA amplification of overlapping DNA fragments using long distance PCR

The design of the primers was based on “classic” criteria such as avoiding regions of secondary structure and self-annealing, aiming for the GC content to be between 40 and 60 % with three C or G at the 3′ of a primer ending to promote binding. The PCR mixture contained 1X PCR buffer, 0.5 μM of each primer, 0.2 mM dNTPs mix and 0.02U.μl^−1^ of Phusion® High-Fidelity DNA polymerase (New England BioLabs). PCR reactions contained 5–10 ng of template plasmid and were carried out under the following conditions: the initial denaturation of 30s at 98 °C, followed by 35 cycles of 10s at 98 C, 30s at 68 °C, 40s per kb at 72 °C, followed by a final elongation of 5 min at 72 °C. All amplified fragments were purified using the PCR clean-up Gel extraction kit (Macherey-Nagel) according to the manufacturer’s instructions and then digested by the *Dpn*I restriction enzyme (1X buffer, 0.5U.μl-1 of *Dpn*I). As *Dpn*I recognizes 5′-GmeATC-3′sites and digests the methylated DNA, it was used to degrade the plasmid template following PCR reactions. A final purification step using the PCR clean-up Gel extraction kit was applied to clean-up PCR products after digestion.

### *In vitro* recombination by Gibson assembly

The three clean-up PCR fragments (F1, F2, mchV, see Fig. 1b) were assembled according to the one-step isothermal DNA assembly method described by Gibson in 2009 [9]: 0.025 pmol of each DNA fragments were pooled in 5 μl, and 15 μl of home -made assembly master mixture according to Gibson’s protocol (500 mM Tris–HCl pH 7.5, 50 mM MgCl_2_, 1 mM dGTP, 1 mM dATP, 1 mM dTTP, 1 mM dCTP, 50 mM DTT, 25 % PEG-8000 and 5 mM NAD) were added to DNAs. The mixture was incubated at 50 °C for 1 h in a thermocycler. In order to control DNA molecules assembly, a 0.8 % agarose gel electrophoresis was performed in 1X TBE buffer, and DNA stained with ethidium bromide (5 μg/mL) for visualization. Three microliters of Gibson assembly reaction were used for transformation of NEB 10-beta Electrocompetent *E.coli* cells according to the manufacturer’s recommendations (New Englands Biolabs). Bacterial plasmid DNA extraction was performed using Wizard® Plus SV Minipreps DNA Purification System kit and protocols (Promega) according to the manufacturer’s instructions. Restriction analysis of the purified plasmids was performed with two different enzymes *Eco*RV and *Hind*III (1X buffer, 1X BSA, 0.5U/μl).

### Plant inoculation

All plants were grown under standard greenhouse conditions (16 h day length; 18–23 °C). To check the infectivity of the FL-cDNA clones, two independent LMVmchVPg_Ec cDNA clones (LV14, LV15) were inoculated as described previously [30] using DNA-coated gold particle bombardment with an Helios® Gene Gun System (Bio-Rad). Each clone was inoculated to six lettuce plants *(Lactuca sativa,* cultivar Trocadéro) in two independent experiments. The upper systemically infected leaves of primarily inoculated by biolistic with the LV15 clone were then used to mechanically inoculate one Trocadéro lettuce and three *Nicotiana benthamiana* plants, in four independent experiments. The infected leaves were ground (ratio 1:4 wt/vol) in a solution of 25 mM Na_2_HPO_4_ containing 0.2 % diethyldithiocarbamate (DIECA) and 100 mg of carborundum.

### RNA extraction and RT-PCR

Total RNAs were extracted from fresh symptomatic systemically infected leaves as previously described [31]. The presence of the mcherry-VPg fusion in the recombinant virus was confirmed by two-step RT-PCR. The reverse transcription was started by mixing 5 μg of total RNAs with 1 μM of oligo (dT) 18 primer and 2 μM of random hexamer primer (N6) in a volume of 12 μl. To disrupt potential secondary structures, the tube was placed 5 min at 65 °C, before adding 8 μl of 10 units RevertAid^TM^ H Minus (Thermo Fisher Scientific Inc.), 1 unit RiboLock™ RNAse Inhibitor (Thermo Fisher Scientific Inc.), 1 X buffer and 1 mM dNTP. The reverse transcription reaction was performed at 25 °C for 10 min, 42 °C for 60 min and 70 °C for 10 min. Following first-strand synthesis, 2 μl of cDNA were transferred to a separate tube for the PCR step involving 2 μM of each of the two primers - P1Hc.fwd and P1Hc.rev - targeting the insertion site of the ectopic mcherryVPg sequence (Table 1), 1X ThermoPol® buffer, 0.25 mM dNTP and 0.05 units Taq DNA polymerase (New England Biolabs). PCR amplification was performed with an initial denaturation step at 95 °C for 2 min, followed by 35 cycles each consisting of 30 s at 95 °C, 30 s at 56 °C and 2 min at 72 °C, and a final step final elongation at 72 °C for 5 min. The amplification products were analyzed on 0.8 % non-denaturing agarose gels and ethidium bromide staining. Their sequence was confirmed by direct sequencing (GATC Biotech Constance, Germany) on both strands.

### Fluorescence and confocal microscopy observations

The mcherry expression at the tissue level was examined by fluorescence microscopy with a 0.8× magnification, using a Leica MZFLIII Fluorescence Stereomicroscope equipped with TXR LP (Texas Red Long Pass) Filter (excitation BP: 540–580 nm), emission LP: 601 nm (Eclipse 800, Nikon, Tokyo, Japan). Subcellular distribution of mCherry fluorescence was analyzed using a Leica TCS SP2 confocal microscope and the LCS Lite Leica software. mcherry fluorescence was assessed with excitation at 543 nm using a Helium–Neon (HeNe) laser, with an emission band of 560–645 nm. The fluorescence of DAPI characteristic of nucleus labelling was assessed with excitation at 40 nm and emission bands of 410 nm to 475 nm.

## Additional files

Additional file 1:
**Cloning by homologous recombination in yeast of an LMVmchVPg full-length cDNA.** **A**. Schematic representation of the *Lettuce mosaic virus* (LMV) LMVmchVPg genome. The mcherry sequence (red star) is fused at the N-terminus of the VPg in the LMV genome. **B**. Schematic representation of the cloning by homologous recombination in yeast. The LMVmchVPg clone is derived from the LMV-0 cDNA clone previously reported by Redondo *et al*. [30] and further modified by Sorel *et al.* [23]. The LMV FL- cDNA was assembled into a pBluescribe-derived vector pBS70T containing an enhanced 35S promoter (P70S), a NOS terminator (NosT), an *E.coli* replication origin (Ori), an ampicillin resistance gene (AmpR), a cassette containing the 2 μ yeast replication origin and a yeast selectable marker (Trp-1promoter and gene, 2 μ-Trp1). The mcherry PCR fragment (mch) was amplified from pmCherry-C1 vector (Clontech) using mch.fwd and mch.rev primers (Additional file 2). The two long distance PCR fragments named A and B (respectively highlighted in green and orange) were amplified by long distance PCR, using the primer pairs (Additional file 2) positioned along the LMV FL-cDNA clone described in Sorel *et al.* [23]. The size of the overlapping regions between the PCR fragments where homologous recombination takes place is indicated. Homologous recombination in yeast of the three fragments was performed according to Sorel *et al*. [23]. bp: base pair; kbp: kilobase pair. Additional file 2:
**Primers used for the LMVmchVPg cloning by homologous recombination in yeast.** The regions of homology allowing recombination in yeast are underlined and indicated in bold. The suffixes “**fwd**” indicate sense primers, while suffixes “**rev**” indicate antisense primers. bp: base-pair, nt: nucleotide. Additional file 3:
**Vein clearing and mosaic symptoms characteristic of LMV infection induced by LMVmchVPg_Ec on lettuce.** **A**. mock-inoculated lettuce. **B**, **C**. vein clearing symptoms (**B**) and mosaic symptoms (**C**) induced on the upper systemically infected leaves at 15 dpi. Additional file 4: Detection by RT-PCR of LMVmchVPg_Ec progeny in systemically infected leaves of lettuce at 15dpi. 0.8 % non-denaturing agarose gel electrophoresis of the reverse transcription-polymerase chain reaction (RT-PCR) amplification products obtained with primers P1Hc.fwd and P1Hc.rev in total RNAs extracts from LMVmchVPg-Ec infected plants (**LV**) or healthy plants (**H)**. Controls: amplification product obtained with the plasmid template LMVmchVPg_Ec (**C+**) or with the plasmid template LMV (without mcherry-VPg) (**C-**). **1 kb+**: 1 Kb Plus DNA Ladder (Invitrogen). The relevant sizes of the expected DNA fragments are indicated at the left. Additional file 5:
**DAPI staining of LMVmchVPg_Ec infected lettuce leaves.** **A**. Fluorescence signals observed in the epidermal cells of the lettuce cultivar Trocadéro using confocal microscopy at 16 dpi. **B**. DAPI staining: the leaf was infiltrated with 0.4 μM/ml diamidino-2-phenylindole (DAPI). The fluorescence of DAPI was assessed with excitation at 40 nm and emission bands of 410 to 475 nm. Scale bars: 16.1 μM (A, B). Additional file 6:
**Flow-chart of the different steps of the procedure.**

## Abbreviations

LMV: *Lettuce mosaic virus*
VPg: Viral protein genome-linked
dpi: Day post inoculation
GA: Gibson assembly
bp: Base pair
FL-cDNA: Full length complementary DNA
RT-PCR: Reverse transcription-polymerase chain reaction
dNTP: Deoxyribonucleotide triphosphate
DTT: Dithiothreitol
NAD: Beta-nicotinamide adenine dinucleotide
TBE: Tris borate EDTA
EDTA: Ethylenediaminetetraacetic acid
SDS-PAGE: Sodium dodecylsulfate polyacrylamide gel electrophoresis
