# Supplementary figures and images for: Gibson assembly: an easy way to clone potyviral full-length infectious cDNA clones expressing an ectopic VPg
Source: Virol J. 2015 Jun 14;12:89. doi: 10.1186/s12985-015-0315-3 (PMC4475333; doi:10.1186/s12985-015-0315-3)

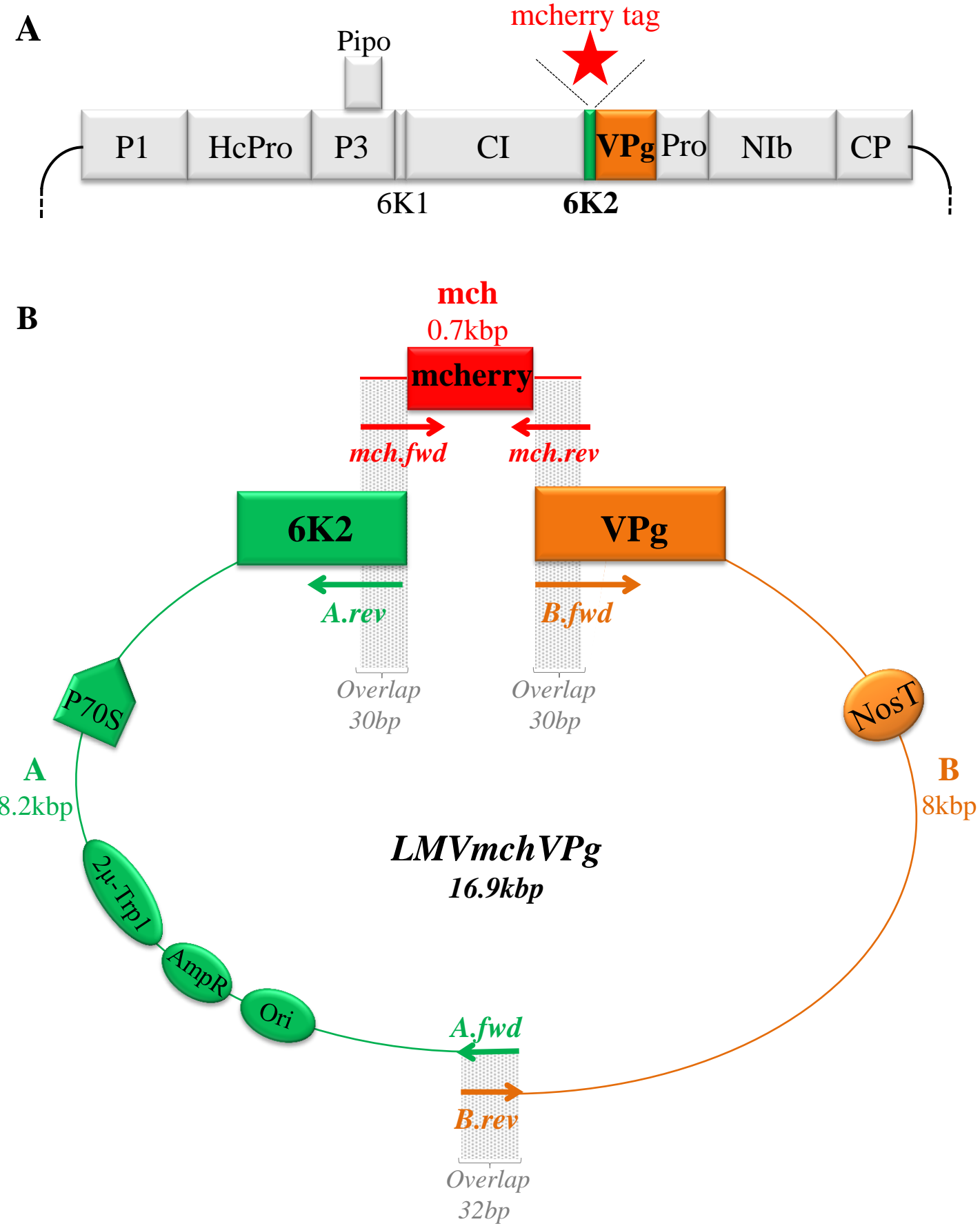

Supplement: Additional file 1: — Cloning by homologous recombination in yeast of an LMVmchVPg full-length cDNA. A. Schematic representation of the Lettuce mosaic virus (LMV) LMVmchVPg genome. The mcherry sequence (red star) is fused at the N-terminus of the VPg in the LMV genome. B. Schematic representation of the cloning by homologous recombination in yeast. The LMVmchVPg clone is derived from the LMV-0 cDNA clone previously reported by Redondo et al. [30] and further modified by Sorel et al. [23]. The LMV FL- cDNA was assembled into a pBluescribe-derived vector pBS70T containing an enhanced 35S promoter (P70S), a NOS terminator (NosT), an E.coli replication origin (Ori), an ampicillin resistance gene (AmpR), a cassette containing the 2 μ yeast replication origin and a yeast selectable marker (Trp-1promoter and gene, 2 μ-Trp1). The mcherry PCR fragment (mch) was amplified from pmCherry-C1 vector (Clontech) using mch.fwd and mch.rev primers (Additional file 2). The two long distance PCR fragments named A and B (respectively highlighted in green and orange) were amplified by long distance PCR, using the primer pairs (Additional file 2) positioned along the LMV FL-cDNA clone described in Sorel et al. [23]. The size of the overlapping regions between the PCR fragments where homologous recombination takes place is indicated. Homologous recombination in yeast of the three fragments was performed according to Sorel et al. [23]. bp: base pair; kbp: kilobase pair. [file 12985_2015_315_MOESM1_ESM.pdf]

**A**

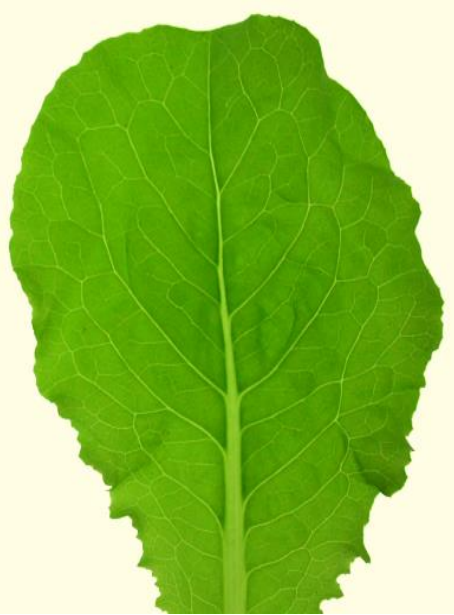

**B**

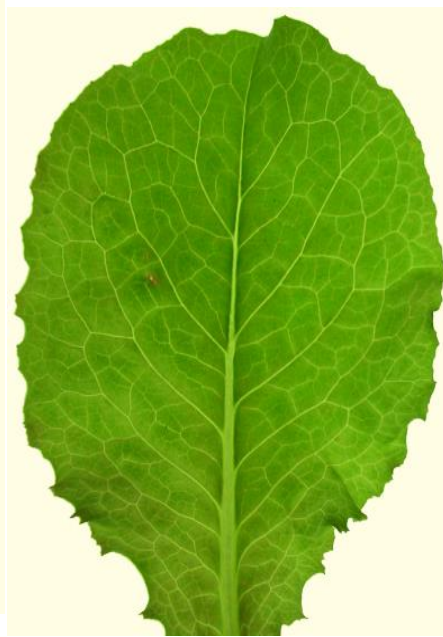

**C**

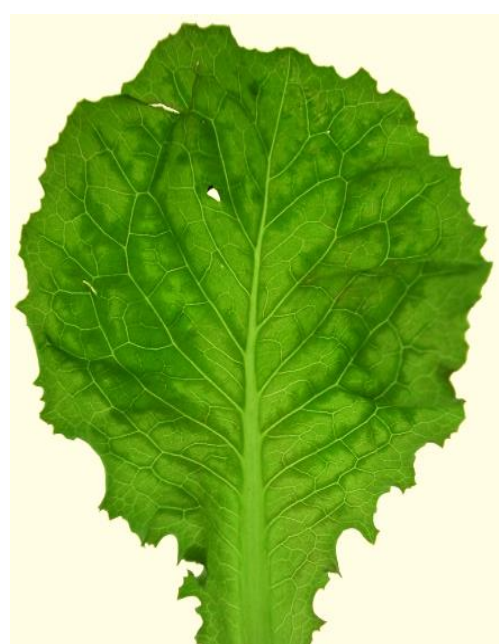

Supplement: Additional file 3: — Vein clearing and mosaic symptoms characteristic of LMV infection induced by LMVmchVPg_Ec on lettuce. A. mock-inoculated lettuce. B, C. vein clearing symptoms (B) and mosaic symptoms (C) induced on the upper systemically infected leaves at 15 dpi. [file 12985_2015_315_MOESM3_ESM.pdf]

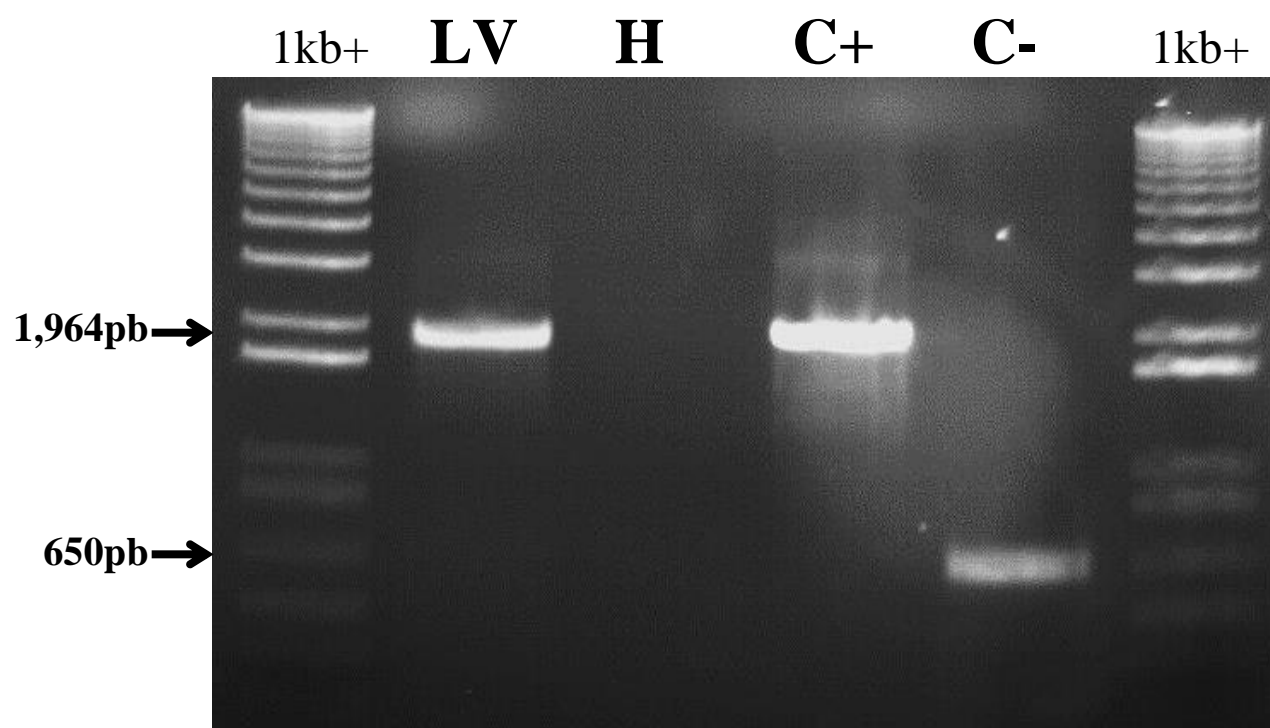

Supplement: Additional file 4: — Detection by RT-PCR of LMVmchVPg_Ec progeny in systemically infected leaves of lettuce at 15dpi. 0.8 % non-denaturing agarose gel electrophoresis of the reverse transcription-polymerase chain reaction (RT-PCR) amplification products obtained with primers P1Hc.fwd and P1Hc.rev in total RNAs extracts from LMVmchVPg-Ec infected plants (LV) or healthy plants (H). Controls: amplification product obtained with the plasmid template LMVmchVPg_Ec (C+) or with the plasmid template LMV (without mcherry-VPg) (C-). 1 kb+: 1 Kb Plus DNA Ladder (Invitrogen). The relevant sizes of the expected DNA fragments are indicated at the left. [file 12985_2015_315_MOESM4_ESM.pdf]

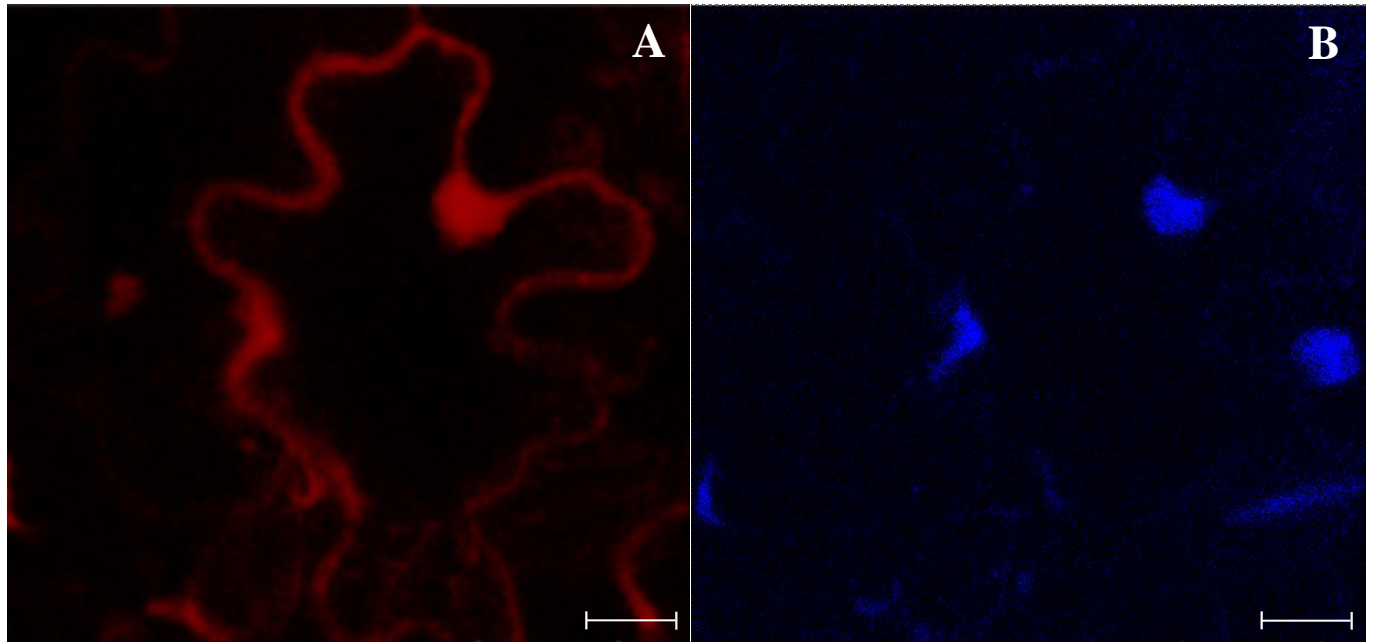

Supplement: Additional file 5: — DAPI staining of LMVmchVPg_Ec infected lettuce leaves. A. Fluorescence signals observed in the epidermal cells of the lettuce cultivar Trocadéro using confocal microscopy at 16 dpi. B. DAPI staining: the leaf was infiltrated with 0.4 μM/ml diamidino-2-phenylindole (DAPI). The fluorescence of DAPI was assessed with excitation at 40 nm and emission bands of 410 to 475 nm. Scale bars: 16.1 μM (A, B). [file 12985_2015_315_MOESM5_ESM.pdf]
