# Supplementary material for: Gibson assembly: an easy way to clone potyviral full-length infectious cDNA clones expressing an ectopic VPg
Source: Virol J. 2015 Jun 14;12:89. doi: 10.1186/s12985-015-0315-3 (PMC4475333; doi:10.1186/s12985-015-0315-3)
Supplement: Additional file 2: — Primers used for the LMVmchVPg cloning by homologous recombination in yeast. The regions of homology allowing recombination in yeast are underlined and indicated in bold. The suffixes “fwd” indicate sense primers, while suffixes “rev” indicate antisense primers. bp: base-pair, nt: nucleotide. [file 12985_2015_315_MOESM2_ESM.pdf]

Primer name	Primer size (nt)	Fragment	Sequence 5'-3'	Product size (kbp)
**mch.fwd**	63	mch	**GACGGGATGGCTGATGCAGTTCGACACCAA**GGCAAAGGTGTGAGCAAGGGCGAGG AGGATAAC	0.7
**mch.rev**	58		**GAGCTTCTGTCTTTGCCGTTTACCTTTGCC**CTTGTACAGCTCGTCCATGCCGCCGGTG	
**B.fwd**	30	B	**GGCAAAGGTAAACGGCAAAGACAGAAGCTC**	8.0
**B.rev**	32		**CATCTATTGCATAGGTAATCTTGCACGTCGCA**	
**A.fwd**	32	A	**TGCGACGTGCAAGATTACCTATGCAATAGATG**	8.2
**A.rev**	30		**TTGGTGTCGAACTGCATCAGCCATCCCGTC**	

Additional file 2. Primers used for the LMVmchVPg cloning by homologous recombination in yeast.

The regions of homology allowing recombination in yeast are underlined and indicated in bold. The suffixes "**fwd"** indicate sense primers, while suffixes "**rev"** indicate antisense primers. bp: base-pair, nt: nucleotide
