# Supplementary material for: Gibson assembly: an easy way to clone potyviral full-length infectious cDNA clones expressing an ectopic VPg
Source: Virol J. 2015 Jun 14;12:89. doi: 10.1186/s12985-015-0315-3 (PMC4475333; doi:10.1186/s12985-015-0315-3)
Supplement: Additional file 6: — Flow-chart of the different steps of the procedure. [file 12985_2015_315_MOESM6_ESM.pdf]

## Step 1 : Long distance PCR of overlapping fragments

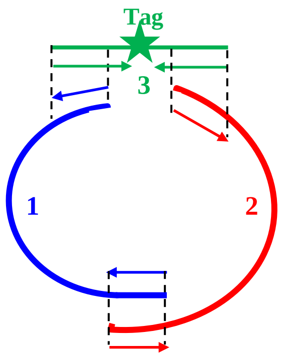


## Step 2 : Gibson Assembly reaction

Example: Fragments 1 and 2

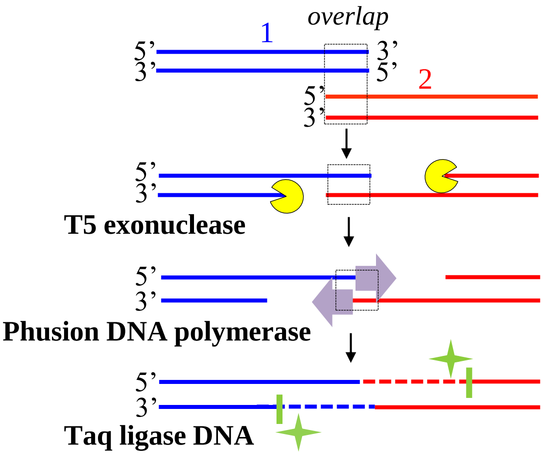


## Step 3 : *E.coli* transformation

## Step 4 : Plasmid extraction

## Step 5 : Plant inoculation
